# Optimizing *Bacillus pasteurii* spore germination and unveiling impermeability mechanisms in microbial self-healing concrete

**DOI:** 10.3389/fmicb.2025.1653557

**Published:** 2025-08-07

**Authors:** Yingying Hu, Weitao Liu, Yutao Zhang, Xuelong Hu

**Affiliations:** ^1^Shandong Key Laboratory of Eco-Environmental Science for the Yellow River Delta, Shandong University of Aeronautics, Binzhou, China; ^2^College of Energy and Mining Engineering, Shandong University of Science and Technology, Qingdao, China; ^3^Key Laboratory of Safety and High-Efficiency Coal Mining, Ministry of Education, Anhui University of Science and Technology, Huainan, China

**Keywords:** self-healing concrete, impermeability, *Bacillus pasteurii*, spore germination, germination conditions, concrete durability

## Abstract

This study investigated the impact of key factors on spore germination of *Bacillus pasteurii*, a self-healing bacterium for concrete, and elucidated its impermeability mechanism to provide theoretical and practical guidance for advanced self-healing concrete development. Controlled experiments determined optimal germination conditions: 2 g/L microcapsule concentration, pH 8, and 1 g/L inosine, yielding peak germination efficiency that highlights parameter synergies. Thermal stimulation for 3 minutes effectively triggered germination, presenting a practical activation approach. MIP and SEM analyses were employed to characterize concrete microstructure. Results showed the alkaline concrete matrix facilitated *B. pasteurii physiology*, while Ca^2+^ had no inhibitory effect, enabling calcium-based additives in formulations. *B. pasteurii*-containing mortar enhanced cement hydration stability; MIP revealed self-healing concrete had an infiltration fractal cone number of 2.832 and trunk fractal dimension of 2.306, similar to conventional materials, indicating no increased structural complexity. Environmental erosion primarily affects 300–10,000 nm pores, pinpointing durability targets. SEM and MIP analyses confirmed *B. pasteurii*-induced vaterite and aragonite calcium carbonate crystals integrated with tobermorite, reducing porosity and enhancing mechanical strength. These findings indicate the bacterium’s potential in self-healing systems, though future research should address complex physicochemical influences and bacterial gradient domestication to improve environmental adaptability.

## 1 Introduction

Concrete, which is the world’s most extensively utilized construction material, is beset with inherent susceptibility to cracking. Plastic shrinkage, thermal stress, and mechanical loading are among the primary factors triggering these cracks (Chen et al., 2010; Dong et al., 2023; Zhao et al., 2022; Shafi, 2022; Xin et al., 2022; Yi et al., 2021; O’Reilly et al., 2022; He et al., 2024; Huang et al., 2024; Guo et al., 2024; Sun et al., 2022; Guo et al., 2025; Thurairajah, 2022; Wang et al., 2025; Liu et al., 2023; Liu et al., 2021). Once cracks form, they act as conduits for water and aggressive agents, hastening the corrosion of reinforcements and the degradation of the structure (Son et al., 2022; Rollakanti and Srinivasu, 2022). Given that cement production contributes 5–7% of global anthropogenic CO₂ emissions (Chen et al., 2010) and that the costs of crack repair are increasing exponentially (Liu et al., 2022), the development of self-healing concrete has become an urgent necessity for enhancing both durability and sustainability in construction.

Microbially induced calcium carbonate precipitation (MICP) has emerged as a promising solution in this context. This approach harnesses bacteria such as *Bacillus subtilis* and *Bacillus pasteurii* to autonomously seal cracks through the biomineralization of calcite (CaCO₃) (Javeed et al., 2024; Nodehi et al., 2022). Alkali-resistant, spore-forming strains demonstrate remarkable resilience in highly alkaline concrete environments (pH > 10). They can reactivate upon water ingress and precipitate CaCO₃ (Zhang et al., 2021; Šovljanski et al., 2022; Zhang et al., 2022; Ramachandran et al., 2001; Zaerkabeh et al., 2022; Jongvivatsakul et al., 2019). Studies have confirmed that optimal bacterial concentrations in the range of 10^5^–10^8^ cells/mL can increase the compressive strength of concrete by up to 42.8% and restore its flexural strength by 14% after cracking (Chen et al., 2010; Javeed et al., 2024). However, several critical challenges impede the widespread application of this technology.

Uncontrolled spore germination remains a significant issue, as it is highly sensitive to variable pH, temperature, and nutrient conditions (Javeed et al., 2024; Nodehi et al., 2022). Additionally, the mechanistic understanding of how MICP-driven crystallization reduces the permeability of concrete and modifies its pore structure is insufficient (Zhang et al., 2021; Qian et al., 2021).

Recent research has highlighted *Sporosarcina pasteurii* as a prime candidate for MICP in concrete. This bacterium exhibits high urease activity and can thrive at pH > 8.5 (Zhang et al., 2022; Ramachandran et al., 2001; Zaerkabeh et al., 2022). Its cell wall, which is rich in peptidoglycan and teichoic acids, serves as an effective nucleation site for CaCO₃. The negatively charged functional groups (such as carboxyl and phosphate groups) on the cell wall can adsorb Ca^2+^ ions, facilitating the precipitation process (Zhang et al., 2022; Qian et al., 2021). Despite these promising characteristics, key parameters governing its germination efficiency, such as germination concentration and thermal activation, as well as the mechanisms underlying its impermeability, such as pore-clogging dynamics and crystallization patterns, remain poorly quantified.

Numerous studies have demonstrated the significant potential of *Bacillus pasteurii* in improving concrete performance through self-healing mechanisms (Akhtar et al., 2025; Cheng et al., 2025; Galano et al., 2025; Ahmad et al., 2025). These studies commonly show that *Bacillus pasteurii*-induced calcium carbonate precipitation can effectively fill cracks, thereby increasing the compressive strength and durability of concrete. For example, research (Akhtar et al., 2025) has shown that incorporating *Sporosarcina pasteurii* into self-compacting concrete (SCC) led to a 16.83–17.31% increase in compressive strength at 120 days compared with that of the control mix. Similarly, studies (Galano et al., 2025) reported that the surface application of *Sporosarcina pasteurii* resulted in an 11–38% increase in the compressive strength of cracked concrete samples. Microstructural analyses via SEM, EDS, and XRD have further confirmed that the precipitated calcite fills pores and cracks, refines the concrete matrix, and improves interfacial bonding (Akhtar et al., 2025; Cheng et al., 2025; Ahmad et al., 2025).

Despite these commonalities, different studies have explored diverse application approaches and focused on various aspects of *Bacillus pasteurii*-based self-healing systems. Studies (Akhtar et al., 2025) have investigated the direct incorporation of *Sporosarcina pasteurii* into SCC at a concentration of 10^5^ cells/mL, highlighting its effectiveness in reducing water absorption (e.g., 1.441% for SCCMSB2 at 28 days vs. 1.848% for the control) and achieving high crack-healing ratios (up to 86.54% at 28 days) for cracks up to 523 μm in size. In contrast, studies (Cheng et al., 2025) emphasized the use of uncoated expanded perlite as a carrier for *Bacillus pasteurii*, revealing that a calcium source concentration of 0.5 mol/L yielded the highest utilization rate (87.65%) and that increasing the self-healing agent dosage enhanced crack-healing effectiveness (e.g., 70.9% healing rate for 1.4–1.6 mm cracks in the B12 group).

One study (Galano et al., 2025) adopted an external application method by spraying *Sporosarcina pasteurii* onto cracked concrete surfaces and demonstrated that this approach could restore compressive strength and achieve effective crack healing, particularly with relatively low bacterial concentrations (≤10^6^). Moreover, studies (Ahmad et al., 2025) focused on *Bacillus subtilis* immobilized in steel slag aggregate (SSA) and reported that a concentration of 10^7^ cells/mL resulted in a 74.2% recovery of compressive strength for 0.65 mm damaged particles and up to 97% strength regain at 28 days, highlighting the potential of SSA as an effective microbial carrier.

Although significant progress has been made in understanding the application of *Bacillus pasteurii* and related species in concrete self-healing, challenges remain. These include improving bacterial viability in the harsh alkaline environment of concrete, optimizing carrier materials and application methods, and evaluating long-term performance under real-world conditions. Further research in these areas is essential to promote the practical application of microbial self-healing technology in sustainable concrete infrastructure. This study aims to investigate the key parameters of *Bacillus pasteurii*-mediated MICP in concrete, with the ultimate goal of developing a more efficient and reliable self-healing concrete system.

## 2 Materials and methods

**Figure 1:**
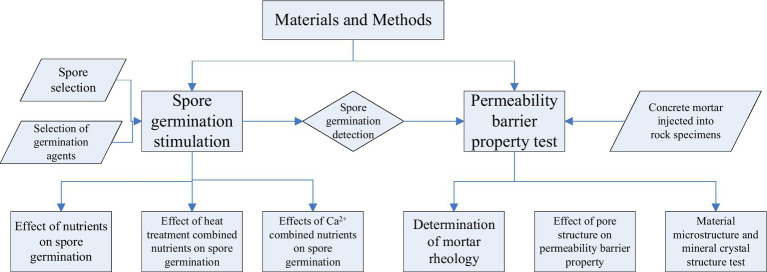
Flow chart of the experimental design.

The germination environment of spores plays a decisive role in subsequent microbial remediation and research on the mechanism of impermeability. Various factors affecting spores have been studied, and the germination time, germination effect, and physiological activity of spores after germination have been determined in different environments, as shown in Figure 1.

### 2.1 Materials

When selecting conditions, the mechanism of spore germination should be considered, and cross-analysis of each factor should be conducted to ensure that the experimental conclusions are correct.

#### 2.1.1 Spore selection

According to the literature (Metwally et al., 2020; Rathore et al., 2021), *Bacillus pasteurii*-treated mixed sand cells can repair cracks within a depth of 3.175 mm. The repair effect of 0.4 mm cracks using ceramic particles, calcium aluminate cement, a silica fume mixture, and diatomaceous earth can reach over 90%, and the bending strength can also be improved by 110%. The width of spore repair cracks in Alkalinistrilicus sp. in expanded clay containing calcium lactate is within 1.0 mm. After surface treatment, *Bacillus cereus* repairs cracks with widths between 0.1 and 0.4 mm, and the tensile strength increases by approximately 30%. The repair crack width of sterile dissolved urea spores containing abundant urea powder is within 0.45 mm (Zhang et al., 2021; Šovljanski et al., 2022; Zhang et al., 2022; Ramachandran et al., 2001; Zaerkabeh et al., 2022). In comparison, *Bacillus pasteurii* has the best repair effect and a certain degree of alkali resistance. Therefore, different sizes of *Bacillus pasteurii* spores were selected for this study.

#### 2.1.2 Selection of germination agents and related experimental materials

Inosine, a nutritional germination agent, has a stimulating effect on spore germination in *Bacillus sphaericus*, *Bacillus cereus*, and other bacteria and has a certain universality (Zhang et al., 2022; Ramachandran et al., 2001; Zaerkabeh et al., 2022; Jongvivatsakul et al., 2019; Qian et al., 2021; Metwally et al., 2020). Therefore, inosine was selected as the germination agent for this experiment.

#### 2.1.3 Concrete mortar injected into rock samples

**Figure 2:**
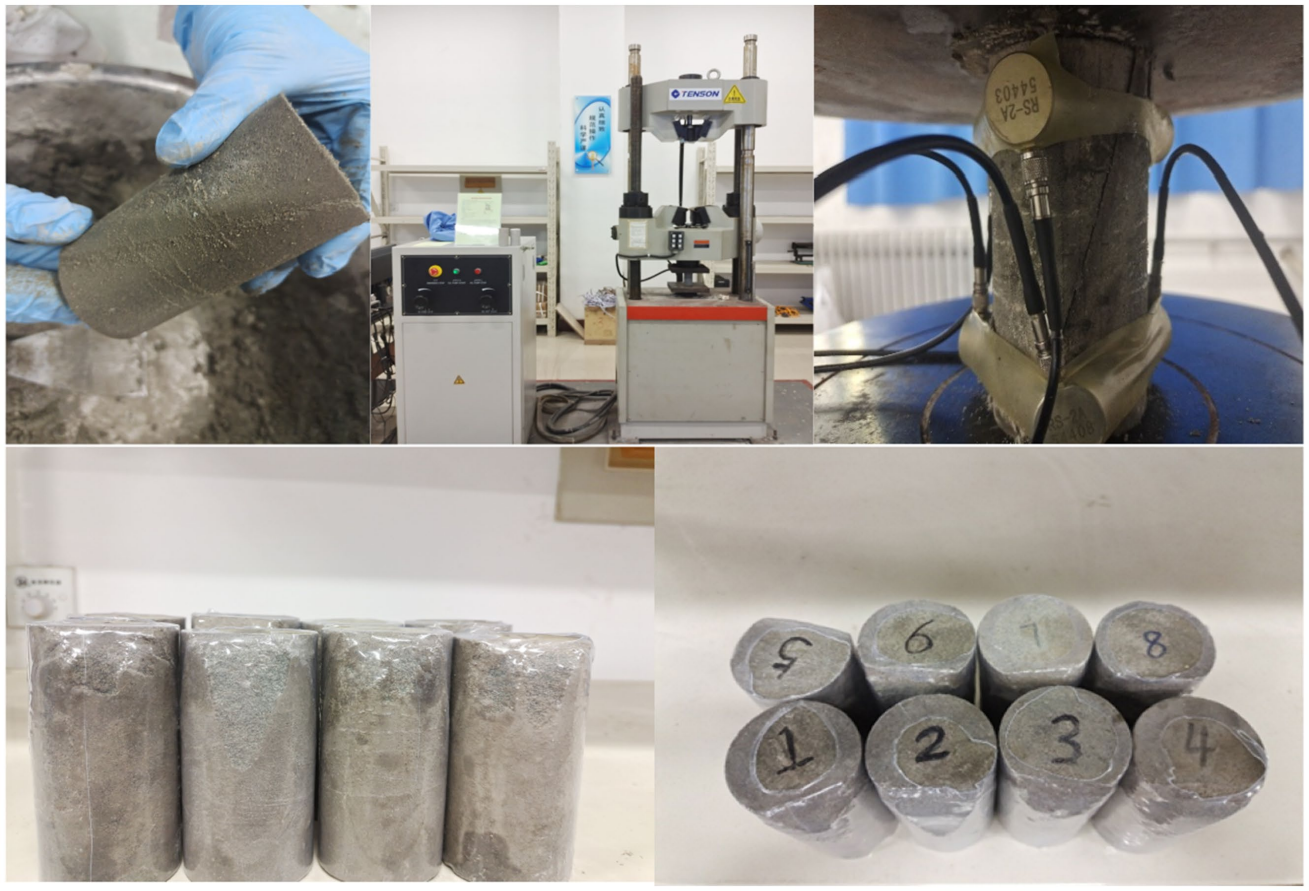
Specimen pressed crack.

On the basis of the results of the spore germination test, 8 groups of samples were designed with dried microcapsules of different dosages and particle sizes, including one blank control group and one fracturing test group, as shown in Table 1. The 8 groups of concrete mortar were injected into the precut rock fractures. After curing for the scheduled age, a universal testing machine was used to prefabricate fractures. The specific operation steps are as follows: the test was carried out on a universal testing machine, where the test specimen to be tested was fixed and installed at the center of the bearing plate, and the disc was adjusted to a stable state. The displacement rate of axial loading was set to 0.01 mm/min, and prefabricated fractures were formed by applying a central concentrated load, as shown in Figure 2.

**Table 1:** The main properties of the concrete samples.

NO.	Microcapsule dosage g/L	pH	w/c ratio	Grade	Dimension (mm)
1	2	7	0.5:1	42.5	100*50
2	4	8	0.5:1	42.5	100*50
3	6	7	0.5:1	42.5	100*50
4	2	8	0.5:1	42.5	100*50
5	4	7	0.5:1	42.5	100*50
6	6	8	0.5:1	42.5	100*50
7	0	/	0.5:1	42.5	100*50
8	0	/	0.5:1	42.5	100*50

### 2.2 Spore repair test

The microcapsules containing spores were ground thoroughly in a mortar to prepare a spore suspension and poured into spore germination medium, where the concentration of inosine in the medium was 2 g/L, the pH was 10, and the mass concentration of Na^+^ was 24 g/L. After 24 h of cultivation in a shaking incubator, the mixture was removed and injected into the purchased rock sample, which was then sealed with plastic film, and the nutrient solution was regularly replaced. The nutrient mixture was prepared at pH 9: urea, 15.62 g/L; yeast extract, 2 g/L; and calcium chloride, 22.5 g/L. The samples were cured for 2 weeks, and the repair effect was observed. The *Bacillus pasteurii* used in this study has a reparative function after germination with inosine.

### 2.3 Spore germination stimulation

A microcapsule suspension with sterilized deionized water was prepared at a concentration of 2 g/L.

#### 2.3.1 Spore germination detection

Typically, the OD_600_ is widely used to monitor bacterial growth because bacterial cells have a strong light absorption capacity at this wavelength, which can effectively avoid interference from commonly used culture media, and there is a good linear relationship between the OD_600_ and bacterial concentration. However, when monitoring microbial spore germination, OD_490_ may be a better choice, mainly for the following reasons. From the perspective of material changes during spore germination, a series of substances are synthesized and decomposed inside the spore during germination. The metabolic products that play a key role in the process of spore germination, such as specific enzymes, coenzymes, or signaling molecules, have stronger characteristic absorption peaks at OD_490_. Owing to its high sensitivity to changes in spore germination-related substances, changes in cell structure and physiological state, absorption of specific pigments, and changes in cell wall structure, OD_490_ can more accurately reflect the process by which spores are gradually activated from a dormant state, initiating metabolism, and eventually forming vegetative cells (Zhang et al., 2022; Ramachandran et al., 2001; Zaerkabeh et al., 2022; Jongvivatsakul et al., 2019).

**Figure 3:**
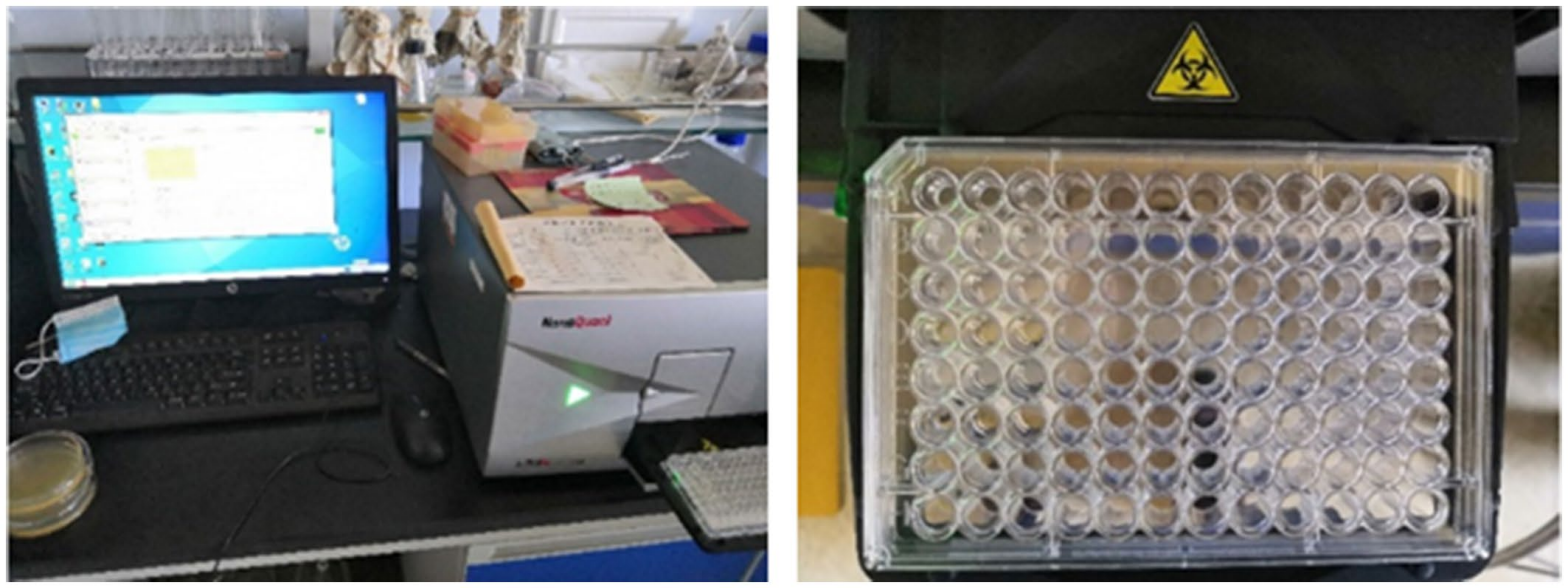
Orifice plate sampling.

The value of the spore suspension at a wavelength of 490 nm was measured with a microplate reader. Formazan can absorb 490 nm light. Formazan exists only in living cells, and the greater the degree of cell activity is, the greater the amount of formazan produced, and the higher the absorbance, which is used to detect cell activity. The cell membrane permeability changed during spore germination. With increasing spore germination rate, the optical density gradually decreases (Javeed et al., 2024; Nodehi et al., 2022; Zhang et al., 2021; Šovljanski et al., 2022). In the experiment, the spore germination state was determined by the change in light density, and the spore germination rate was calculated. The specific process was as follows: A spore capsule made of 2 grams of E-51 epoxy resin was prepared as a suspension and added to the prepared germination culture medium. After shaking, the bacterial mixture was transferred to a 96-well polyethylene plate with a 200 μL pipette gun. The OD_490_ value (optical density) was measured every hour with a multifunctional microplate reader (Figure 3).

#### 2.3.2 The effects of nutrients on spore germination

**Figure 4:**
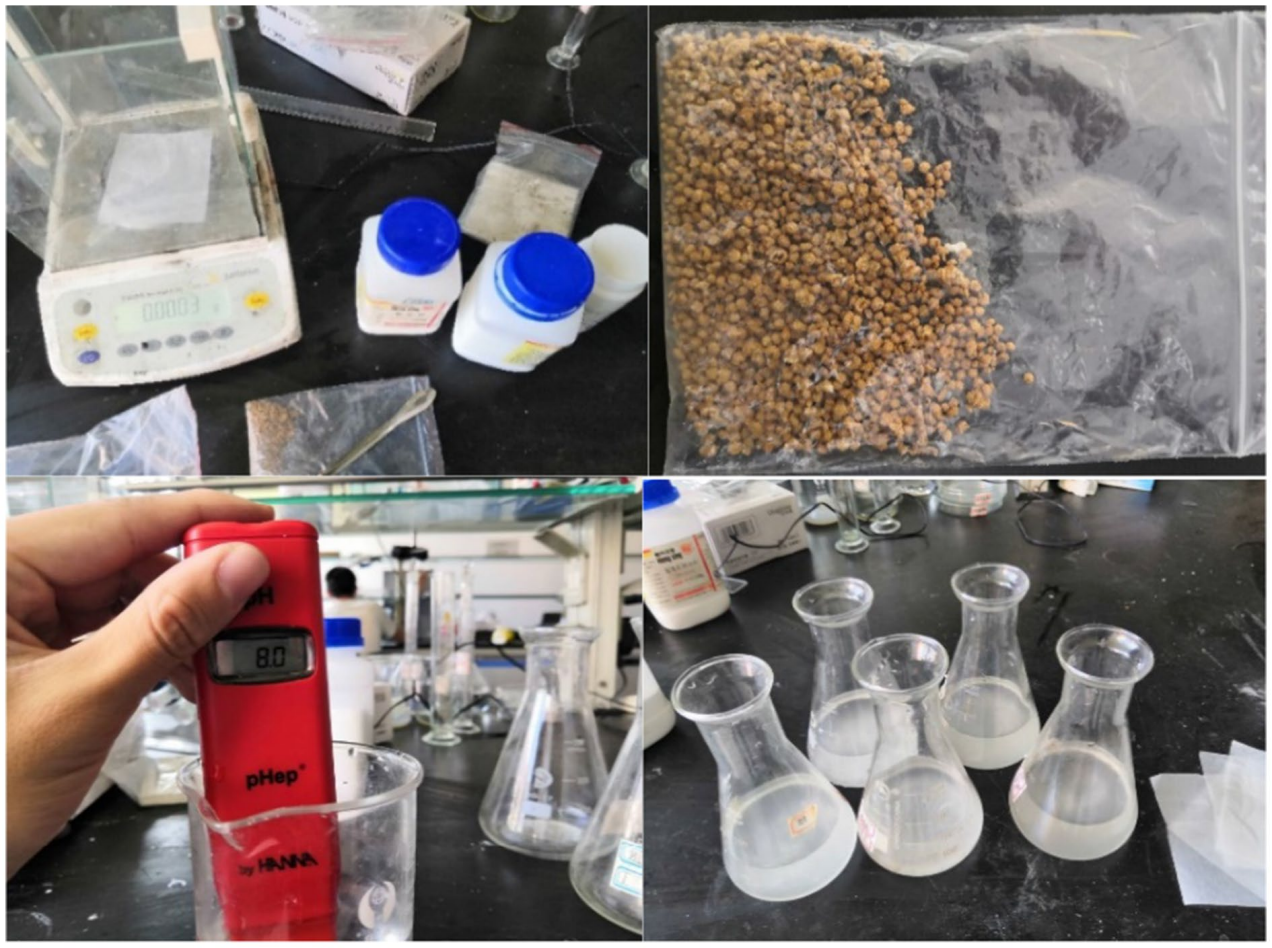
Experimental procedure.

The germination culture medium was configured, and inosine was selected as the germination agent. Four groups of experiments were set up, as shown in Table 2. The pH of Group A was adjusted to 8, and the concentration of inosine was 0.5 g/L. The pH of Group B was adjusted to 9, and the concentration of inosine was 1 g/L. The pH of Group C was adjusted to 9, and the concentration of inosine was 2 g/L. Compared with that of group B, the effect of the inosine concentration on spore germination was investigated. The pH of Group D was adjusted to 11, and the concentration of inosine was 2 g/L. Compared with that of group C, the effect of high-concentration pH on germination was investigated. After synthesizing groups B, C and D, group A was analyzed. After 5 min, the absorbance was measured. In the early stage of spore germination, the germination agent enters the spore core, the permeability of the spore membrane increases, and the formazan disappears, which leads to a decrease in the refractive index. This process was measured by the loss of absorbance at 490 nm. The OD_490_ value of the bacterial mixture was detected every hour after the start of the test. Figure 4 shows some instruments and reagents used in the experiment and the experimental process.

**Table 2:** Experimental design.

Group	pH	Na^+^ concentration (g/L)	Inosine concentration (g/L)
A	8	4.0	0.5
B	9	4.0	1
C	9	4.0	2
D	11	4.0	2

The OD value was used as the dependent variable, and the pH and inosine concentration were selected as the influencing factors. The average value of each factor was obtained at three levels, and the experiment was carried out. The experimental design is shown in Table 2.

#### 2.3.3 Effects of heat treatment combined with nutrient addition on spore germination

At present, research on spore germination has focused mostly on improving the conditions of nonphysical and chemical factors, but there are few reports on the physical and chemical factors of spore germination (Zaerkabeh et al., 2022; Jongvivatsakul et al., 2019; Qian et al., 2021; Metwally et al., 2020; Rathore et al., 2021). In this experiment, the appropriate conditions were selected for the first experiment. The spores were placed in a constant-temperature water bath at a set temperature of 50°C for 1, 3, 5 or 10 min and then cooled to room temperature. The OD_490_ value was detected every hour according to the configuration of the above group C solution. The effect of heat shock time on spore germination was investigated, and the change in the OD value during spore germination was observed, which was repeated three times.

**Figure 5:**
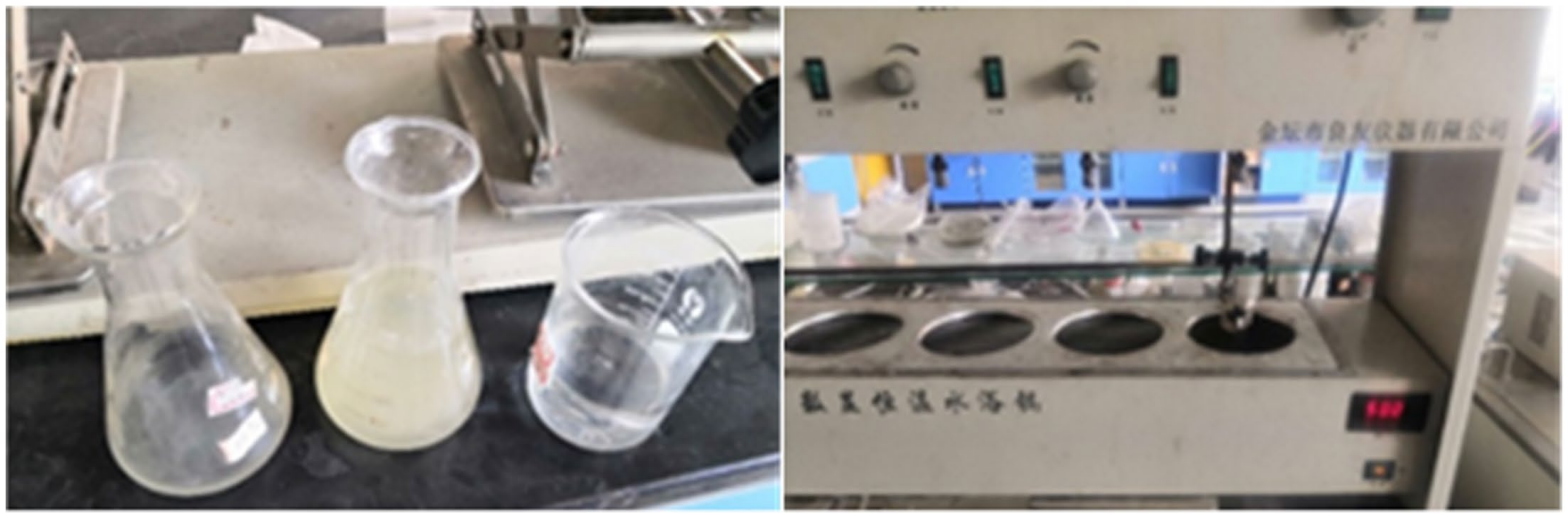
Spore solution to be heat treated.

The solution of group C was prepared with a Na^+^ mass concentration of 4 g/L and a pH of 9. Each group was heated for 1, 3, 5, or 10 min, and each group was tested three times. The C0 group was used as the control group to investigate the effect of thermal stimulation alone on germination. The experimental design is shown in Table 3. Figure 5 shows the experimental instruments and reagents used in the experiment.

**Table 3:** Experimental design.

Group	Inosine concentration (g/L)	Heating time (min)
C0	0	3
C1	2	1
C2	2	3
C3	2	5
C4	2	10

#### 2.3.4 Effects of Ca^2+^ combined with nutrients on spore germination

In an experiment in which *Bacillus sphaericus* was used to repair concrete, the presence of Ca^2+^ inhibited spore germination. When inosine is used as the germination agent, 10.5 g/L Na^+^ can increase the germination rate of spores to more than 75%, while the germination rate of spores containing 0.6 g/L Ca^2+^ is only 6%, but a high concentration of Na^+^ can eliminate the inhibitory effect of Ca^2+^ on spore germination (Zaerkabeh et al., 2022; Jongvivatsakul et al., 2019; Qian et al., 2021; Metwally et al., 2020). In the internal environment of concrete, the content of Ca^2+^ is very high, which is an important factor in investigating the conditions of spore germination. Therefore, the effect of Ca^2+^ on the germination of pasteurized spores was investigated via group C experiments.

### 2.4 Permeability barrier property test of the embedded microbial capsule mortar

#### 2.4.1 Determination of mortar rheology

**Figure 6:**
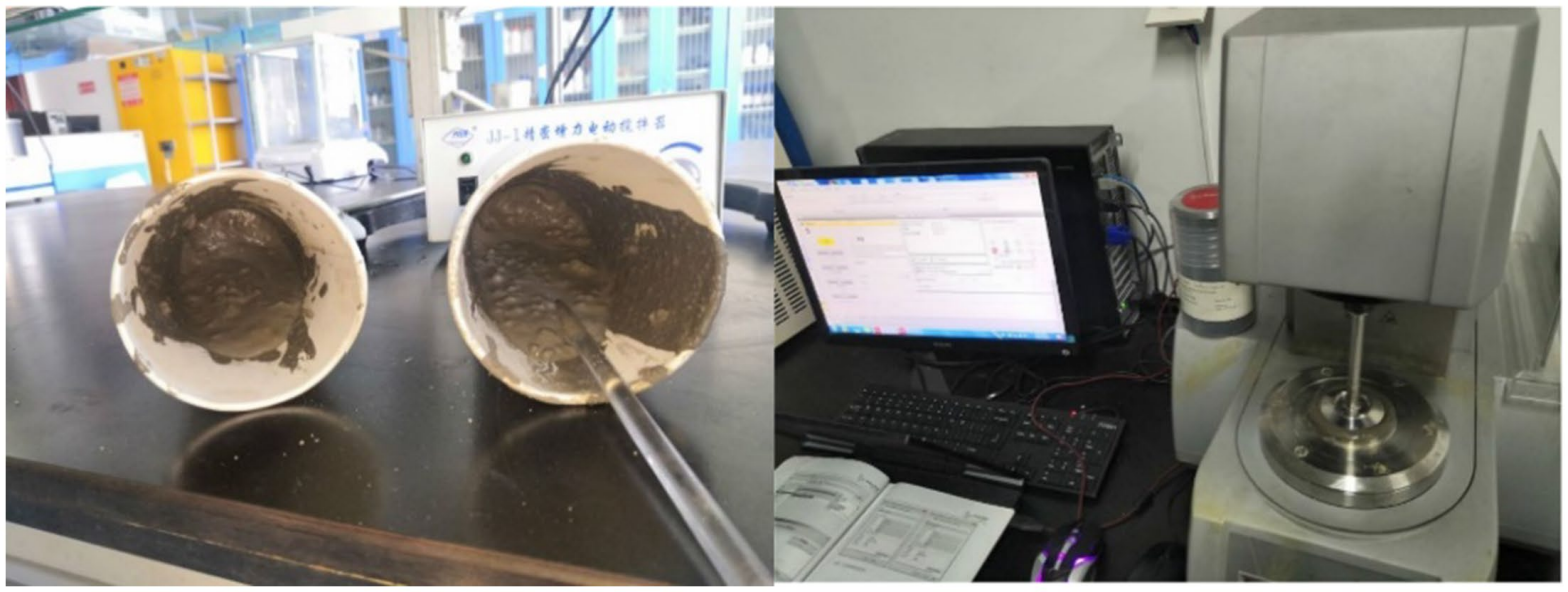
Rheometer test process.

After the sample was weighed according to the mass ratio of cement:water:sand = 1:0.5:1.64, it was poured into a mixing basin for full mixing to make a mortar. The stirred mortar was divided into two parts, one of which was supplemented with 300 μm diameter *Bacillus pasteurii* microcapsules coated with epoxy resin with a total mass of 4%, and stirring was continued. The resulting mortar is shown in Figure 6.

The rheological test of the microbial mixed mortar was then carried out as follows: ① Shear test: First, the rheometer was initialized, the temperature was set to room temperature (25.3°C), the gap was adjusted to zero, the cone plate was selected for contact shear, the test distance was adjusted to 2.75 mm, and the variable shear rate was selected from low to high $\dot{\gamma }$ =0–100 s^−1^. Sixteen data points were selected for variable rate shear. ② Oscillation test: After the sample was presheared for $\dot{\gamma }$ =100 s^−1^, 30 s, the shear strain *γ* = 0.5% and angular frequency *ω* range of 0.1–100 were set, and 16 data points were selected for the oscillation test.

#### 2.4.2 Effect of the pore structure on the permeability barrier property

Studies (Liu et al., 2021; Son et al., 2022; Rollakanti and Srinivasu, 2022; Liu et al., 2022) have shown that the surface layer of microbial self-healing concrete is often attached to a layer of MICP (secretion of self-healing bacteria), forming a mineralized layer composed of tightly structured cements, which can effectively reduce the absorption of capillary water and reduce the permeability of the material. This property allows the self-healing mortar used for wall structure reinforcement to maintain the water vapor difference between the internal and external environments, effectively preventing the alkaline corrosion of capillary water on the concrete structure and improving the durability of the masonry structure. To study the microstructure of microbial self-healing concrete and explore its pore size distribution, the mercury pressure method was selected for related detection. When mercury is pressed into concrete, mercury enters the pores of the material. When the pressure generated by the mercury porosimeter is greater than the capillary pressure at the pore pipe, mercury is pressed into the capillary pore. The pressure of the mercury porosimeter on mercury is equal to the pressure on the capillary at that time. The pore pipe radius can be determined according to the capillary pressure formula pc = 2cosθ/r. The volume of mercury in the pore is equal to the pore volume connected by the throat, and the specific surface area can be obtained. The relationship between the capillary wall pressure and the pore pipe radius can be obtained by continuously changing the pressure generated by the mercury intrusion porosimeter.

As a liquid metal at room temperature, mercury can penetrate into concrete because of its liquid form, its shape changes after being pressed into the interior, and its electrical conductivity also changes. During mercury intrusion experiments, mercury is continuously pressed into pores of different shapes, and the electrical signals generated by the sensor to detect mercury are also constantly changing. The generated electrical signals are converted into data by a computer for data processing, and the analysis images are obtained. The computer calculates and analyses various types of data related to porosity.

The pore size distribution of self-healing concrete samples mixed with 4% microcapsules was tested via an Auto Pore V9600 mercury porosimeter. The pressure used in the test was increased from 0.10 ps to 61000.00 ps, and the contact angle was 130.000°. By measuring the volume of the mercury metal pressed into the sample, the connected pores of the material were measured twice.

#### 2.4.3 Material microstructure and mineral crystal structure test

To analyze the relationship between the microbial mineral composition and the crystal structure formed in microbial mortar and its strength, SEM analysis was conducted on the calcium carbonate precipitates secreted by microorganisms in microbial cement mortar, as well as on cement with capsules and the fractured surfaces of cement containing microcapsules.

## 3 Results

### 3.1 Spore repair test

ImageJ software was used to perform binarization processing on the images of the sample cracks, obtaining binarized images of the cracks and the number of pixel points. By analyzing the changes in the binarized images and applying the area repair rate formula to the pixel points, the changes in the repair effect during the sample curing process can be reflected (Nodehi et al., 2022; Zhang et al., 2021; Šovljanski et al., 2022; Zhang et al., 2022; Ramachandran et al., 2001). The area repair rate formula is shown in Equation (1):


(1) $$\text{Area repair rate}=\frac{S_{0}-S_{t}}{S_{0}}\times 100\%$$

*S_0_*: Number of pixels before curing.
*S_t_*: Number of pixels at the curing age t.

**Figure 7:**
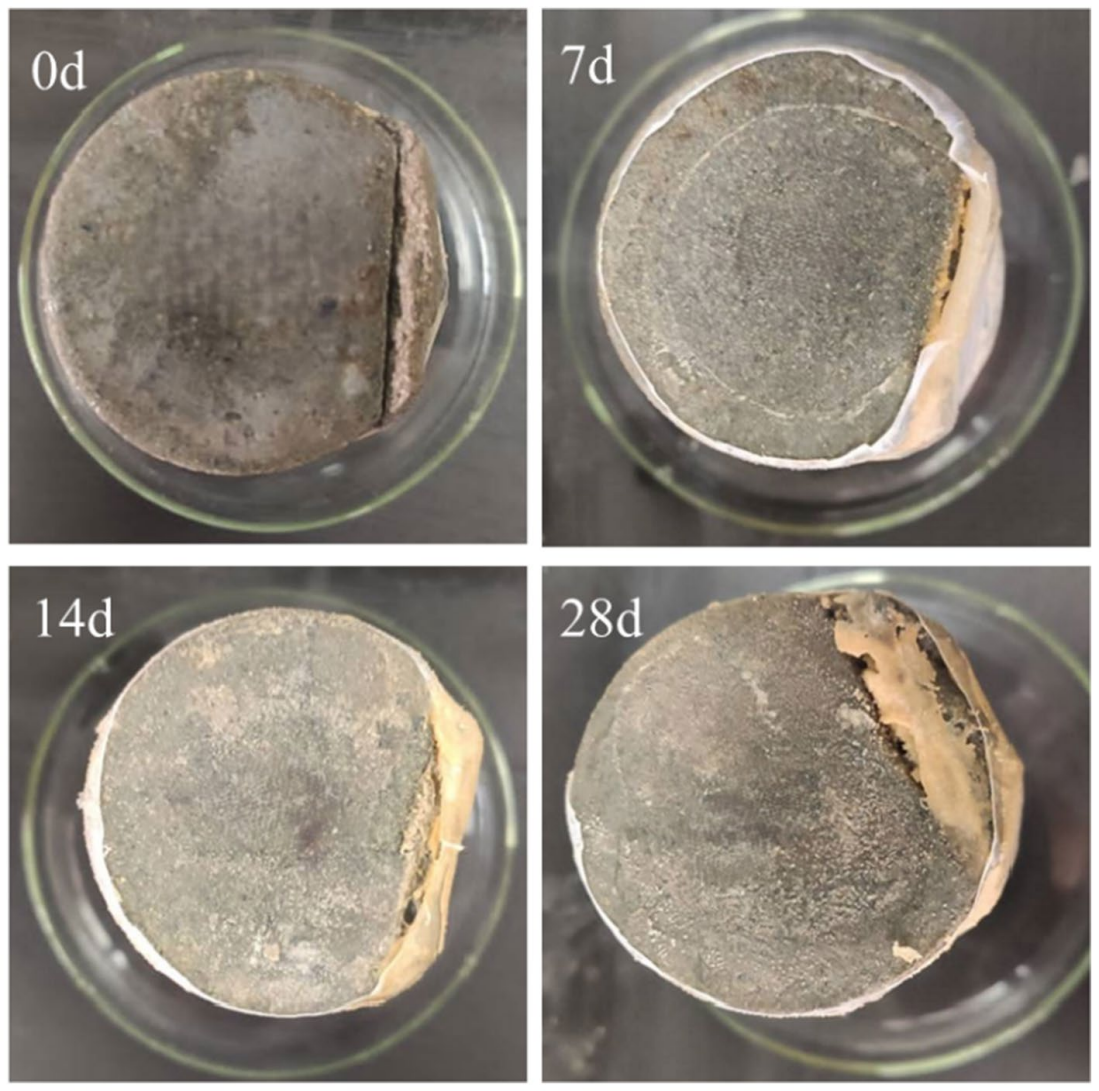
Evolution of cracks with increasing curing age under bacterial activity.

Under the action of a germination agent with an inosine concentration of 1 g/L, the sample with a microcapsule dosage of 2 g/L and a pH of 8 presented the best repair effect. The development and evolution diagrams of its repair at 0 d, 7 d, 14 d, and 28 d are shown in Figure 7. An examination of the cracks revealed that the use of inosine as a germination agent promoted the germination of *Bacillus pasteurii*, and the germinated spores were able to mineralize.

### 3.2 Effects of nutrients on spore germination

**Figure 8:**
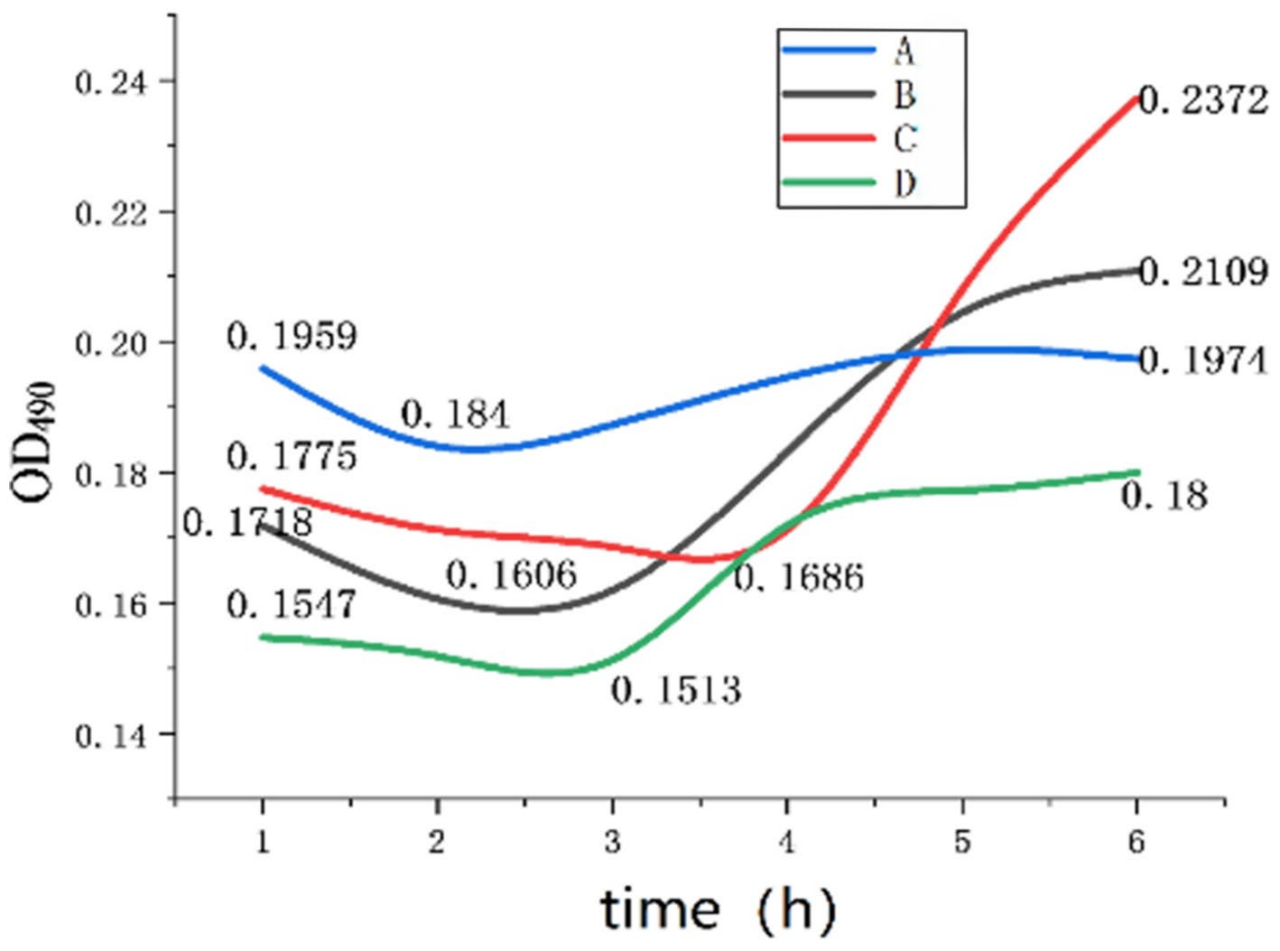
OD values during germination.

In the absence of heat treatment, the mass concentration of Na^+^ was 4.0 g/L. Inosine was used as a germination agent to determine the OD_490_ value of the spore germination process, and Excel and Origin were used for statistical analysis, as shown in Figure 8.

As shown in Figure 8 and Table 2, Group A represents the germination culture medium with a pH of 8, a Na^+^ concentration of 4 g/L, and an inosine concentration of 0.5 g/L; Group B represents the one with a pH of 9, a Na^+^ concentration of 4 g/L, and an inosine concentration of 1 g/L; Group C represents the one with a pH of 9, a Na^+^ concentration of 4 g/L, and an inosine concentration of 2 g/L; and Group D represents the one with a pH of 11, a Na^+^ concentration of 4 g/L, and an inosine concentration of 2 g/L.

Figure 6 shows the curve of Group A. When the spores were cultured in an environment with an inosine concentration of 0.5 g/L and a pH of 8, the OD value decreased within 1 to 2 h, indicating that spore germination occurred within 2 h. The B group curve revealed that when the spores were cultured in an environment with 1 g/L inosine and a pH of 9, the OD value decreased within 1–2.5 h, indicating that spore germination was completed within 2.5 h of germination. After completion, the nutritional bacteria begin to carry out corresponding physiological activities, the OD value increases significantly, and then, there is a gradual stabilization trend.

According to the curve of group C, when the spores were cultured in an environment with 2 g/L inosine and a pH of 9, the OD value decreased within 1–4 h, and the amplitude was relatively gentle, indicating that the germination of the spores was relatively gentle. After germination, it becomes a nutrient cell, and the OD begins to significantly increase cell activity. According to the D group curve, when the spores were cultured in an environment with 2 g/L inosine and pH 11, the OD value decreased within 1 to 3 h, indicating that spore germination was completed within 3 h. After the germination of the spore is complete, it becomes a vegetative cell, and the physiological activity quickly stabilizes.

According to the experimental data, compared with those of groups B and C, the germination rate decreased when the inosine concentration in these experiments was between 1 g/L and 2 g/L, indicating that the optimal inosine concentration for germination was less than 1 g/L. After germination, the physiological activity of the nutritional cells in group C increased, possibly because inosine, as a nutrient, provides a certain amount of energy for the physiological activities of nutritional cells. According to Group C and Group D, excessively high pH inhibits spore germination, or a high alkali environment changes the nature of inosine, and the generated salt affects the physiological activities of vegetative cells to a certain extent. Finally, compared with Group A, low pH was more conducive to spore germination, but it was not conducive to the physiological activities of vegetative cells. The optimum concentration of inosine for germination was between 0.5 g/L and 1 g/L.

### 3.3 Thermal stimulation combined with nutrients

After heating, a microplate reader was used for detection immediately after there was no water vapor effect, after which the OD value was detected every hour, and the data were continuously recorded. The experimental data analysis results are shown in Figure 7.

**Figure 9:**
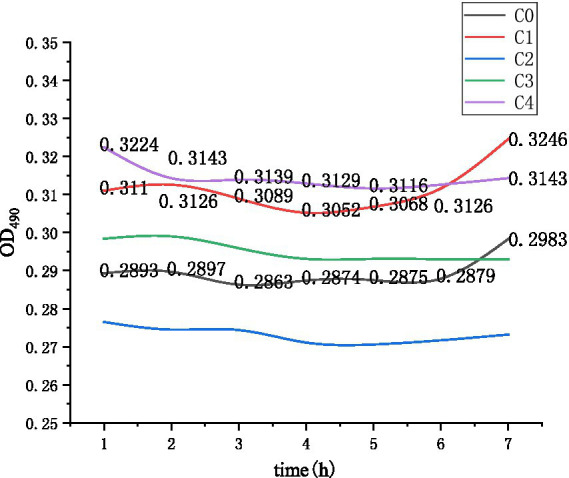
Changes in the OD values during germination after thermal stimulation.

As shown in Figure 9 and Table 3, C0 represents the germination solution with an inosine concentration of 0 g/L and a heating time of 3 min; C1 represents the germination solution with an inosine concentration of 2 g/L and a heating time of 1 min; C2 represents the germination solution with an inosine concentration of 2 g/L and a heating time of 3 min; C3 represents the germination solution with an inosine concentration of 2 g/L and a heating time of 5 min; and C4 represents the germination solution with an inosine concentration of 2 g/L and a heating time of 10 min.

As shown in the C1 curve in Figure 9, the OD value decreased within 2 to 4 h after the spores were heated for 1 min, indicating that spore germination occurred within 2 to 4 h and that physiological activity was increased after germination. The OD values of curves C2, C3 and C4 did not change significantly, and the spores failed to germinate. The spore mixture represented by curve C0 does not contain a germination agent, but the spores can also germinate, indicating that thermal activation can stimulate the related receptor proteins on the spore coat or spore cortex, thereby stimulating spore germination.

According to the experimental results, Figures 8, 9 are compared and analyzed. When the Na^+^ mass concentration was 4.0 g/L and the pH was set to 9, the effect of spore germination after heat treatment worsened, indicating that *Bacillus pasteurii* may have a related defense mechanism for the superheated environment or a synergistic effect of inosine in the heated and alkaline environment. Chemical reactions produce other substances, which affect spore germination and affect the overall OD value. The spores can also germinate under heat treatment without a germination agent at 50°C.

### 3.4 Effect of Ca^2+^ on spore germination

**Figure 10:**
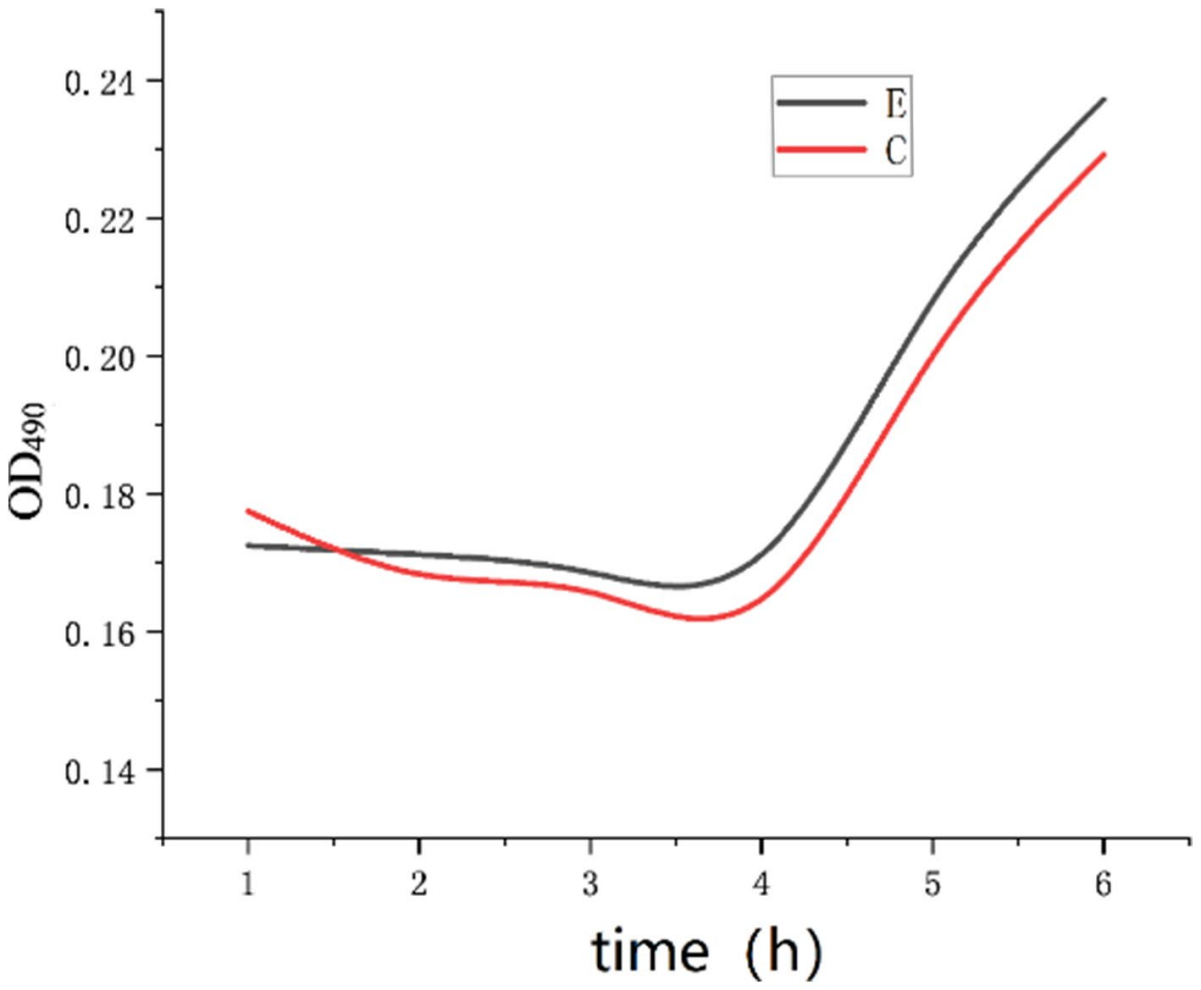
OD curve with Ca^2+^ added.

The experimental results of group E were compared with those of group C. The mass concentration of Na^+^ was 4.0 g/L, the inosine concentration was 2 g/L, the pH was 9, and calcium chloride was added such that the concentration of Ca^2+^ in the solution was 1 g/L. The above experimental tests were repeated three times to record the OD_490_ values, and the experimental results were compared with those of group C, as shown in Figure 10.

As shown in Figure 10, there was no significant change in the OD_490_ of the E group curve compared with that of the C group curve. *Bacillus pasteurii* can germinate within 4 h. Specifically, under the conditions of 4.0 g/L Na^+^, 2 g/L inosine and pH 9, 1 g/L Ca^2+^ had no significant effect on the germination of *Bacillus pasteurii*. It may also be that the concentration of 4.0 g/L Na^+^ in the solution relieved the inhibition of Ca^2+^.

Ionic interactions are crucial. Compared with a high-Na^+^ environment, the availability of Ca^2+^ increases under low-Na^+^ conditions, but the risk of toxicity also increases. The coexistence with other cations, especially K^+^ and Mg^2+^, can significantly alter the mode of action of Ca^2+^, and Mg^2+^ is generally a strong antagonist. The types of anions, such as Cl^−^, SO₄^2−^, and HPO₄^2−^, also affect the formation of Ca^2+^ precipitates. From the perspective of ionic interactions, in an environment with a relatively low Na^+^ concentration, Na^+^ may not be able to fully occupy the relevant ion-binding sites on the surface of spores, resulting in a decrease in the ability of spores to respond to germination signals. In addition, a lower Na^+^ concentration may affect the spore membrane potential and the function of ion channels, hindering the transmembrane transport of substances inside spores, which in turn affects key steps in the spore germination process, such as the release of Ca^2+^-DPA (calcium dipicolinate) and the activation of germination-specific lytic enzymes.

In an extremely low-Na^+^ background, an excessively high Ca^2+^ concentration may lead to an unexpected increase in external osmotic pressure, which may instead be detrimental to sufficient hydration and expansion of the core in the later stages of germination. Therefore, at relatively low Na^+^ concentrations, the promoting effect of Ca^2+^ on the germination of *Bacillus pasteurii* spores depends on the specific concentration of Ca^2+^, the types and concentrations of other coexisting ions in the environment, and the overall osmotic pressure balance. The competitive, synergistic, and antagonistic effects of ions significantly differ under different salt conditions.

The germination conditions of *Bacillus* were explored. The outermost layer of spores is the spore coat, which is an important structure for maintaining the stress resistance of spores. The surface layer is composed of proteins deposited from damaged toxic proteins, which also blocks the possibility of protein nutrients stimulating germination. Studies (Liu et al., 2022) have shown that the spore coating of *Bacillus subtilis* can protect spores from the killing effect of some small-molecule chemical reagents, but the mechanism is not clear.

The inner layer of the spore coat is the spore cortex, which occupies a large volume and is composed of two layers of peptidoglycan. Its structure is loose and important for maintaining the low water content of spores. The inner peptidoglycan will not change much even if the spore germinates as a vegetative cell structure. After germination, it becomes the spore wall of the vegetative cell. During the germination of spores, the cortex is hydrolyzed under the action of the lytic enzyme secreted by the spore, but it does not hydrolyze the inner spore wall in the cortex. Some researchers have observed that cortical peptidoglycan is hydrolyzed by heat treatment via electron microscopy. Moreover, high pressure and heat treatment (HTPS) greatly reduce the activation energy of the peptide bond hydrolysis reaction and increase the speed of the reaction. Some scholars speculate that high pressure and high temperature promote the nonenzymatic hydrolysis of peptides, but research on this mechanism is based mainly on killing spores, which also has certain reference significance in germination. In the study of the effects of ultrahigh pressure combined with physical and chemical factors on the peptidoglycan content of the spore cortex, the germination efficiency of the nutrient combination was greater than that of the single nutrient germination agent, indicating that as long as the relevant defense mechanism was destroyed, enough stimulation was applied to the corresponding protein receptors to allow spores to germinate (Zaerkabeh et al., 2022; Jongvivatsakul et al., 2019; Qian et al., 2021; Metwally et al., 2020; Rathore et al., 2021).

How to stimulate the corresponding germination receptors through the cortex and spore coat or by using the cortex and spore coat is the key to promoting the germination of spores.

In this study, when inosine was used as a germination agent, the optimum pH for germination was less than 9, and the concentration of inosine was less than 1 g/L. When the pH was 1 and the inosine concentration was 0.5 g/L, germination could be completed in approximately 2 hours. When the concentration of inosine exceeded 1 g/L, the germination rate decreased with increasing concentration. When the pH reached 11, the germination and physiological activities of the cells were inhibited. A short heat treatment can also promote spore germination, but the germination rate is lower than that at 6 h, and germination is not complete. When the pH was 9 and the concentration of inosine was 2 g/L, germination was delayed for approximately 1 h after heat treatment for 1 min. The spores did not germinate after heat treatment for more than 3 min, possibly because of the denaturation of inosine caused by heating in an alkaline environment for too long or because the spores had a related defense mechanism against a superheated environment. The presence of Ca^2+^ had no significant effect on the germination of *Bacillus pasteurii*. However, Ca^2+^ can still affect the later stages of *Bacillus pasteurii*, such as bacterial activity, metabolism, and mineralization. An appropriate amount of Ca^2+^ can promote the activity of *Bacillus pasteurii*. Adding calcium ions in advance can help cells maintain osmotic pressure balance and promote the growth of *Bacillus pasteurii*. Ca^2+^ can act as a cofactor for various intracellular enzymes, participating in physiological processes such as intracellular signal transduction and material transport, thereby maintaining the normal physiological functions of cells and improving bacterial activity. High concentrations of Ca^2+^ may compete with other metal ions, interfering with the absorption and utilization of essential metal ions by bacteria and thus inhibiting bacterial activity.

Ca^2+^ has a significant effect on the metabolism of *Bacillus pasteurii*, especially in the process of inducing calcium carbonate precipitation. *Bacillus pasteurii* contains urease, which can decompose urea into NH₃ and CO₂. The bacterial surface carries negative charges and continuously binds to cations (Ca^2+^) in the solution. In this process, the concentration of Ca^2+^ has a significant effect on urease activity. A suitable concentration of Ca^2+^ helps maintain the active conformation of urease and promotes the catabolism of urea. From the perspective of metabolic pathways, Ca^2+^ may be involved in processes related to urease synthesis, transport, or regulation in bacteria.

*Bacillus pasteurii* plays a core role in the mineralization process. This provides nucleation sites for the formation of calcium carbonate crystals and accelerates the precipitation efficiency of calcium ions. On the one hand, the concentration of Ca^2+^ affects the yield and quality of mineralization products. When the concentration of Ca^2+^ in the environment is appropriate, the bacterial surface adsorbs sufficient Ca^2+^, and after urease decomposes urea to produce carbonate ions, calcium carbonate precipitation can be efficiently generated. On the other hand, Ca^2+^ affects the crystal structure and morphology of mineralization products (Rathore et al., 2021; Akhtar et al., 2025; Cheng et al., 2025; Galano et al., 2025; Ahmad et al., 2025).

### 3.5 Analysis of the microscopic experimental results

#### 3.5.1 Rheological test results

As a suspension dispersion system, mortar is a non-Newtonian fluid. Viscoelasticity is the main property affected by the interaction force between the internal suspension dispersed particles and the viscosity of the slurry (Shafi, 2022; Rollakanti and Srinivasu, 2022). The particles in the system are mixed with the solution to form a complex network structure mixed with multiple forces. When slurry yield strain occurs, the interior is composed mainly of interparticle cohesion and interparticle friction.

**Figure 11:**
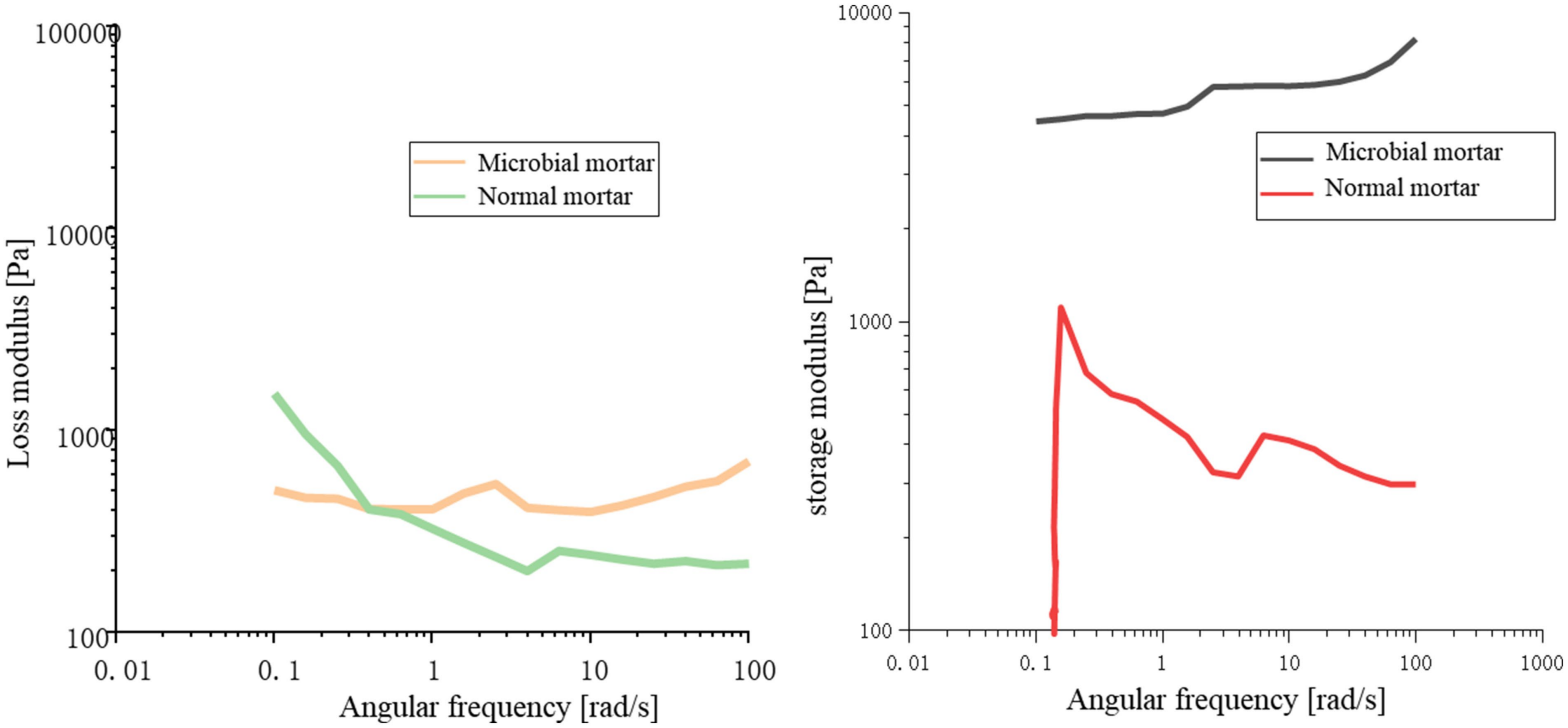
Comparison of the loss modulus and storage modulus.

From Figure 11, it can be concluded that compared with ordinary mortar, microbial mortar has greater energy storage at the same angular frequency; that is, the yield stress of microbial mortar is greater than that of ordinary mortar. With increasing angular frequency, in a short period of time, the loss modulus and storage modulus of the microbial mortar are basically unchanged, and the initial stage of the ordinary mortar is slightly reduced. This may be because the viscosity of ordinary mortar is not sufficient, resulting in water analysis affecting its overall properties.

**Figure 12:**
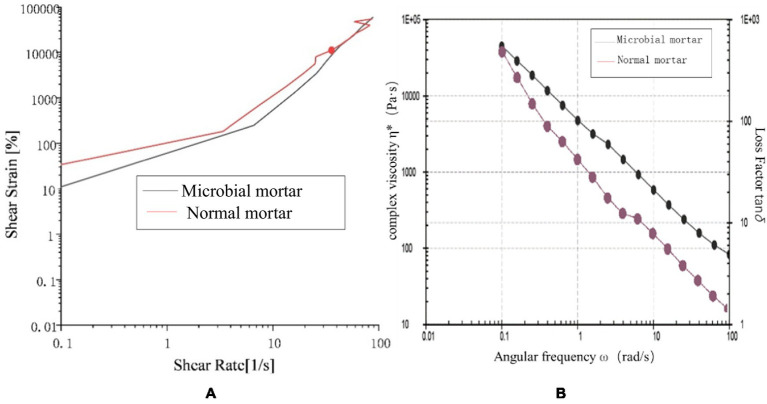
Rheological properties: **(a)** shear strain comparison and **(b)** composite viscosity comparison between the two types of mortar.

Figure 12 shows that the shear strain of ordinary mortar is always greater than that of microbial mortar with increasing shear rate, and the turning point of the slope of the broken line is earlier, which is the same as the conclusion that the yield stress of microbial mortar is greater in Figure 11. A comparison of the data in Figure 12 reveals that the composite viscosity of the microbial mortar is relatively high, which is in line with the above conclusion. The reason may be that with the addition of microbial capsules, owing to the presence of epoxy resin, the slurry is more evenly wrapped with suspended particles to stabilize the adsorption force on the surface of the particles, and the liquid becomes more viscous (Guo et al., 2024; Sun et al., 2022; Guo et al., 2025; Thurairajah, 2022; Wang et al., 2025; Liu et al., 2023; Liu et al., 2021).

#### 3.5.2 Pore test results

The pore radius can be measured via the relationship between the capillary pore radius and mercury intrusion pressure via mercury intrusion porosimetry (MIP), as shown in Equation 2. The volume of mercury intruded into pores of different sizes per unit mass of the sample varies with pressure. By adjusting the pressure of the porosimeter and measuring the mercury intrusion volume at different pressures, the porosity of the tested sample can be calculated.


(2) $$R_{p}=\frac{-2\text{σcosθ}}{P}$$

where *R_p_* is the pore radius (mm), *σ* is the surface tension coefficient of liquid mercury (dynes/cm or mN/m), *θ* is the contact angle between mercury and the sample (degrees), and Pin is the mercury intrusion pressure (psi).

The experimental results indicate that the total permeation volume of the sample under a pressure of 2992.38 psi is 0.0487 mL/g, and the total pore area is 1.488 m^2^/g. After microbial grouting, the average pore diameter (4 V/A) decreased to 130.82 nm, and the proportion of small pores significantly increased. The bulk density at 0.50 psi was 2.32 g/mL, and the measured apparent density of the microbial grouting sample was 2.61 g/m^3^, which is higher than the common apparent density range of sandstones in coal mines. These findings indicate that the injection of microbial capsules reduced the water absorption rate of the rock sample without weakening its compressive strength.

The pore structure analysis revealed that the permeability of the sample is 434.6 md, the permeability fractal dimension is 2.83, and the backbone fractal dimension is 2.3. The internal pore surface area and pore distribution uniformity of the cement sample can be evaluated via the fractal dimension; a higher fractal dimension indicates poorer pore distribution uniformity and insufficient compressive performance. Testing of the pore structure revealed that, upon comparison, the fractal dimensions of the microbial grouting materials and various conventional cement materials were not significantly different. The addition of an appropriate proportion of microbial capsules did not significantly affect the compressive strength of the cement matrix. After being injected into rock fractures, the microbial grouting material effectively provided normal strength reinforcement.

**Figure 13:**
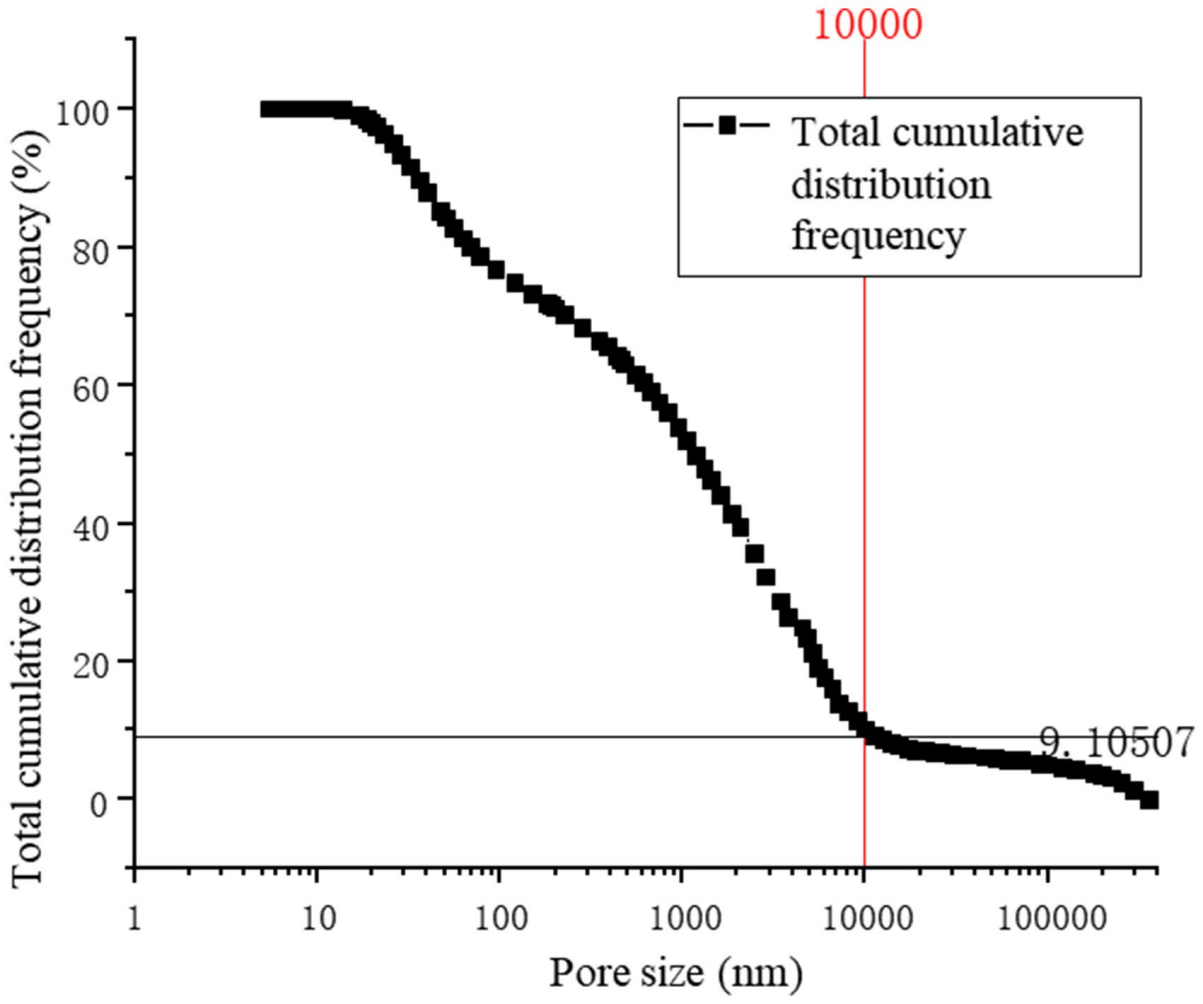
Total cumulative distribution frequency.

**Figure 14:**
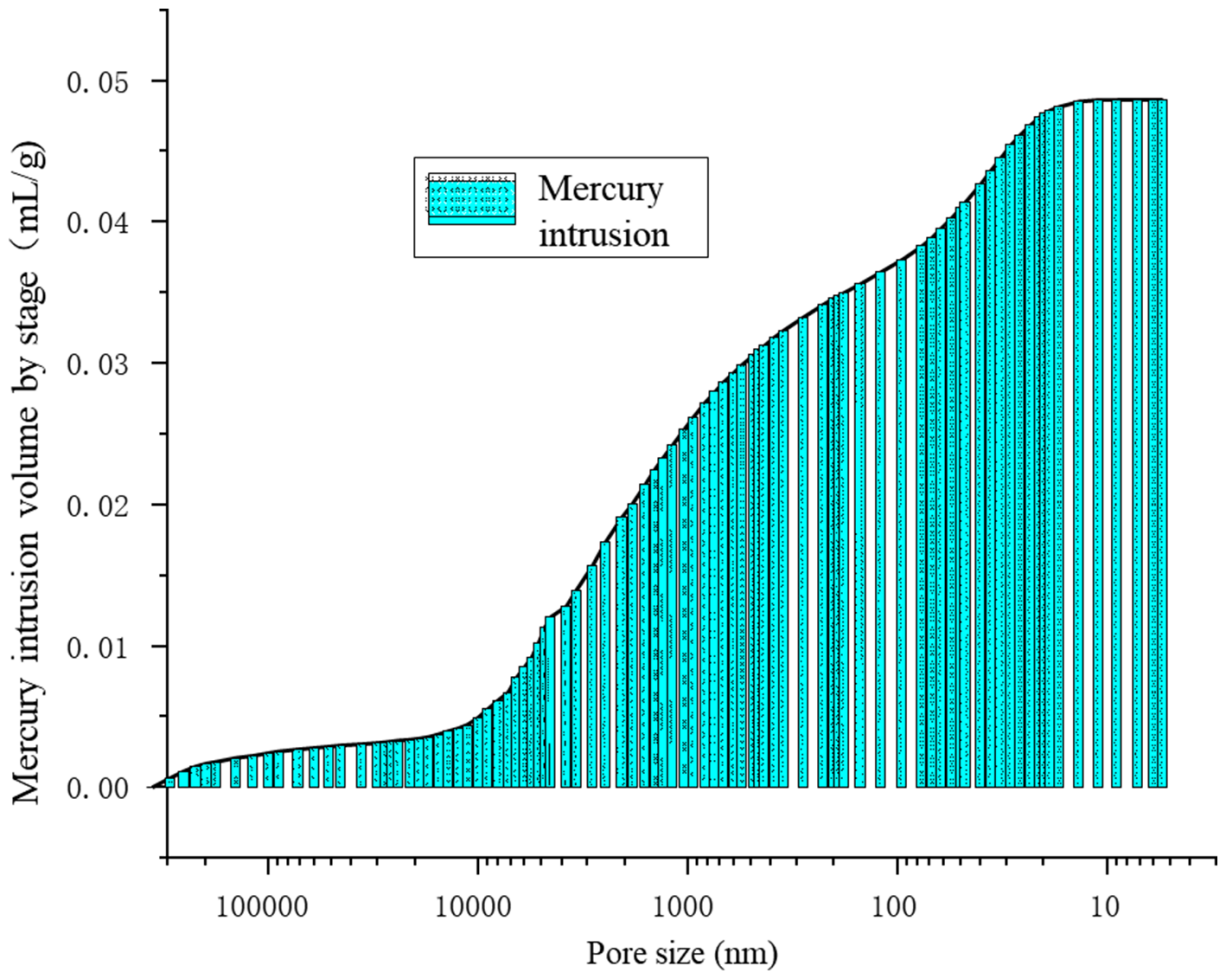
Mercury intrusion volume by stage.

**Figure 15:**
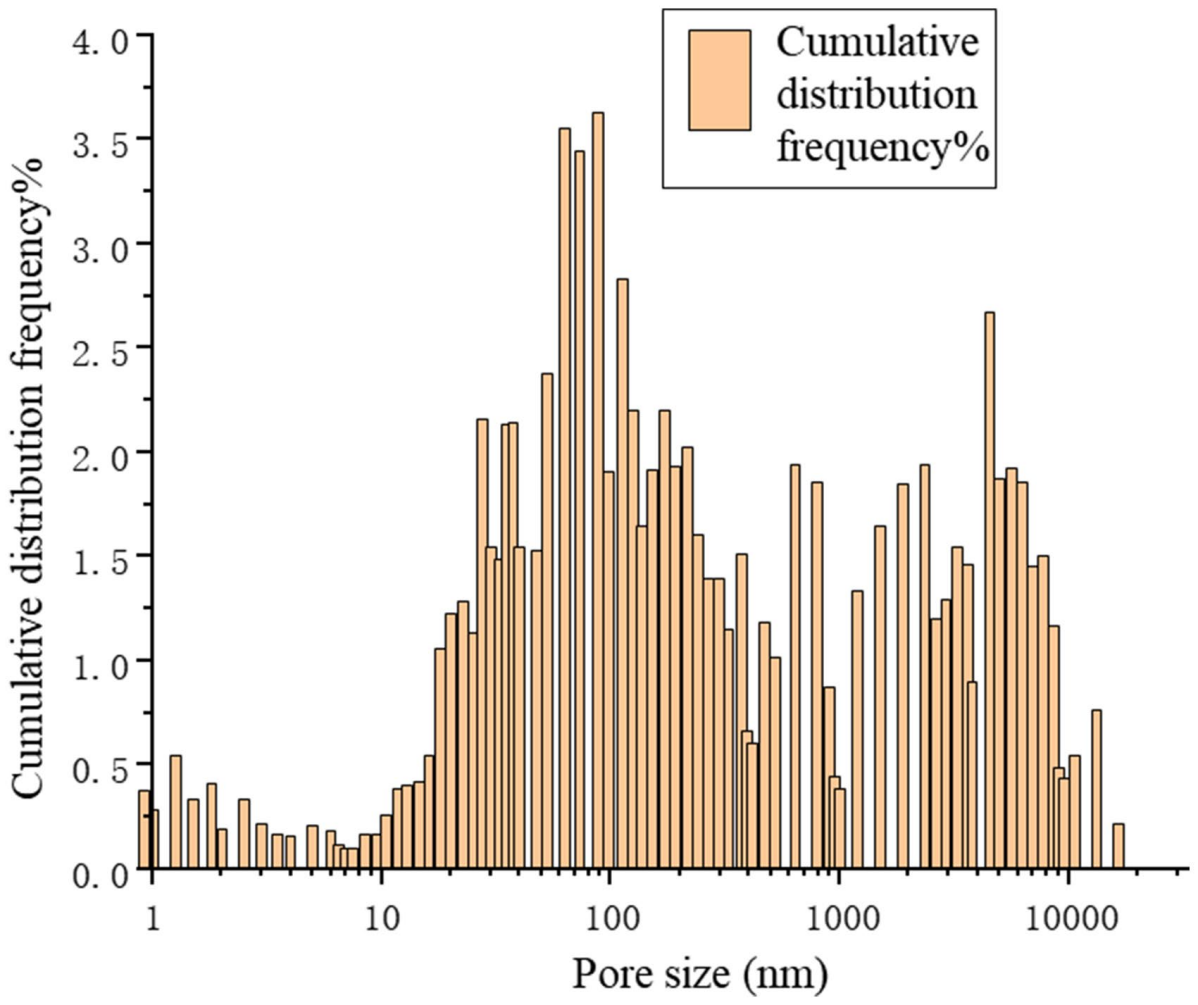
Cumulative distribution frequency.

Figure 13 provides a precise localization of the cumulative distribution frequency in Figures 14, 15. From the relationship curve between the pore diameter and mercury intrusion volume, it can be concluded that during the mercury intrusion process, mercury is primarily concentrated in micropores with diameters smaller than 10 μm. This indicates that pores larger than 10 μm account for only 9.1% of the internal pore structure of the sample.

**Figure 16:**
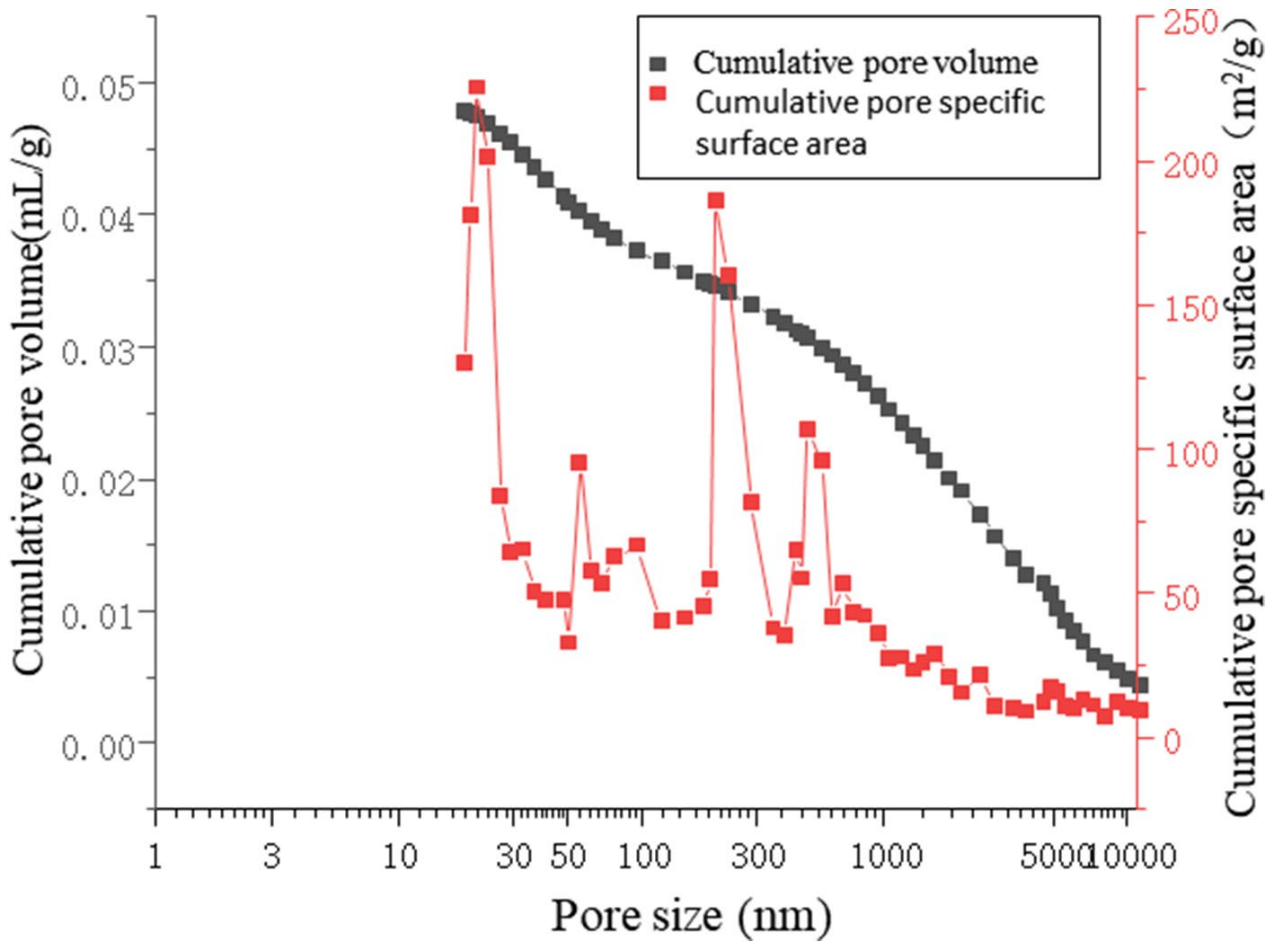
Aperture distribution analysis chart.

As shown in Figure 16, the pore volume is primarily concentrated in the range of 300–10,000 nm. For the microbial grouting samples, the slope of the cumulative pore volume curve is lower in the 100–300 nm range than in the 10–100 nm range. The specific surface area of the pores remains stable in the 30–300 nm range, indicating that the pores in this region are well defined and isolated, making them less prone to water adsorption. In the 300–10,000 nm range, the slope is notably steeper in the 1–10 nm segment, suggesting that the pore distribution is predominantly within the 300–10,000 nm range. This implies that changes in the specific surface area are attributed mainly to pores in this size range. Consequently, it can be concluded that in microbial grouting samples, the adsorption, condensation, and diffusion of air and water primarily occur within pores measuring 300–10,000 nm (Jongvivatsakul et al., 2019; Qian et al., 2021; Metwally et al., 2020). The measured interstitial porosity was 25.95%, with a breakthrough pressure ratio of 8.32. The porosity of the microbial grouting sample was 11.29%, which is relatively low compared with that of conventional sandstone samples. These findings indicate that the pore structure of the rock sample was refined after microbial grouting remediation. This is because the crystal pores formed by MICP are at the nanoscale. During the formation of microbial grout pores, the precipitation and layered coating of calcium carbonate crystal particles are anchored by bacteria, resulting in a high degree of uniformity and facilitating the formation of a dense structure. These crystals adsorb onto existing pores and establish stable cementation with rock particles, creating effective cohesion between the crystals and mineral grains. The consolidated sedimentation and the crystal arrangement under water–rock interactions reduce interparticle porosity, thereby decreasing the hydraulic connectivity and permeability of the rock layer. The irregular pore shapes contribute to increased porosity, which is caused by the interconnectedness of the pores.

#### 3.5.3 SEM

**Figure 17:**
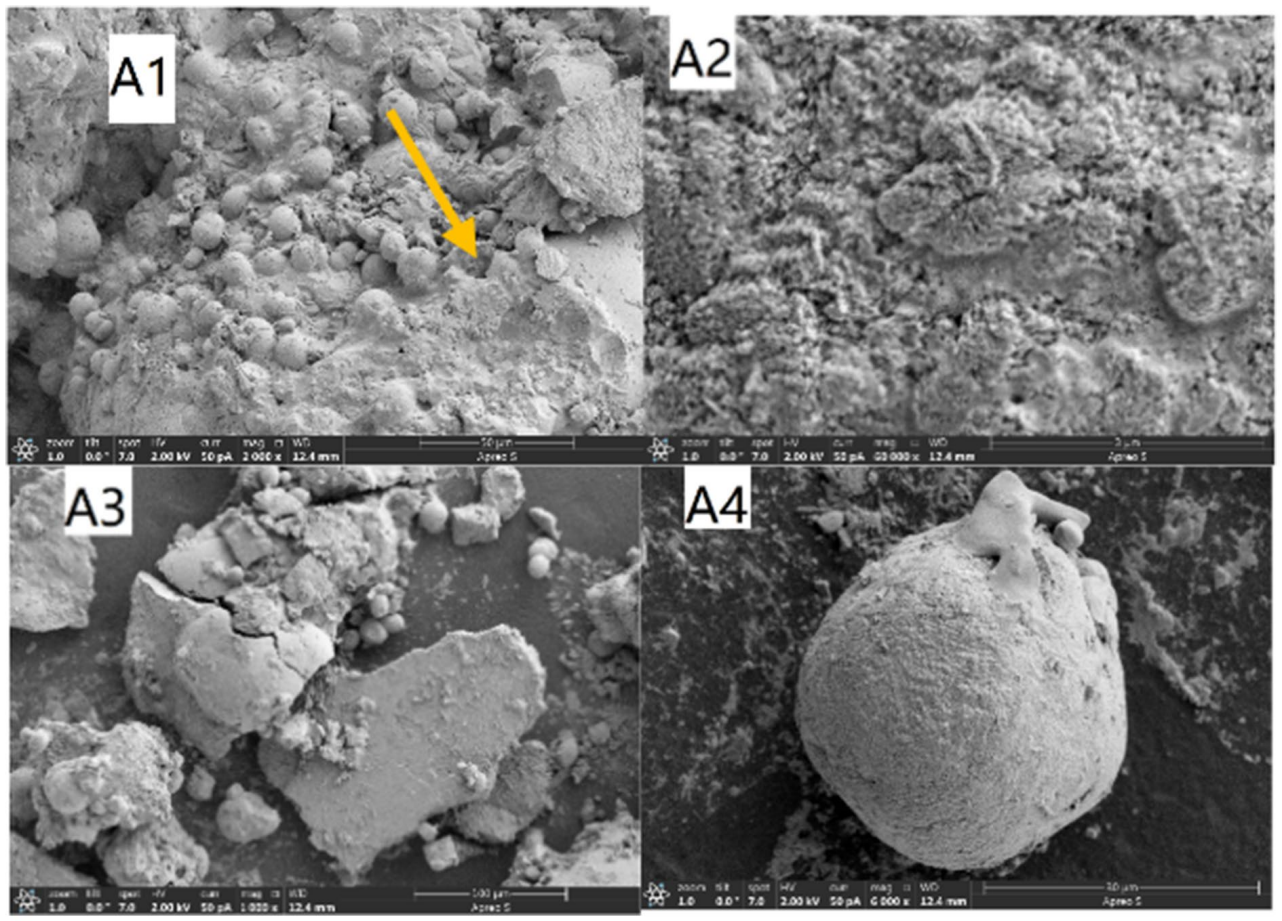
Microbial precipitation products.

**Figure 18:**
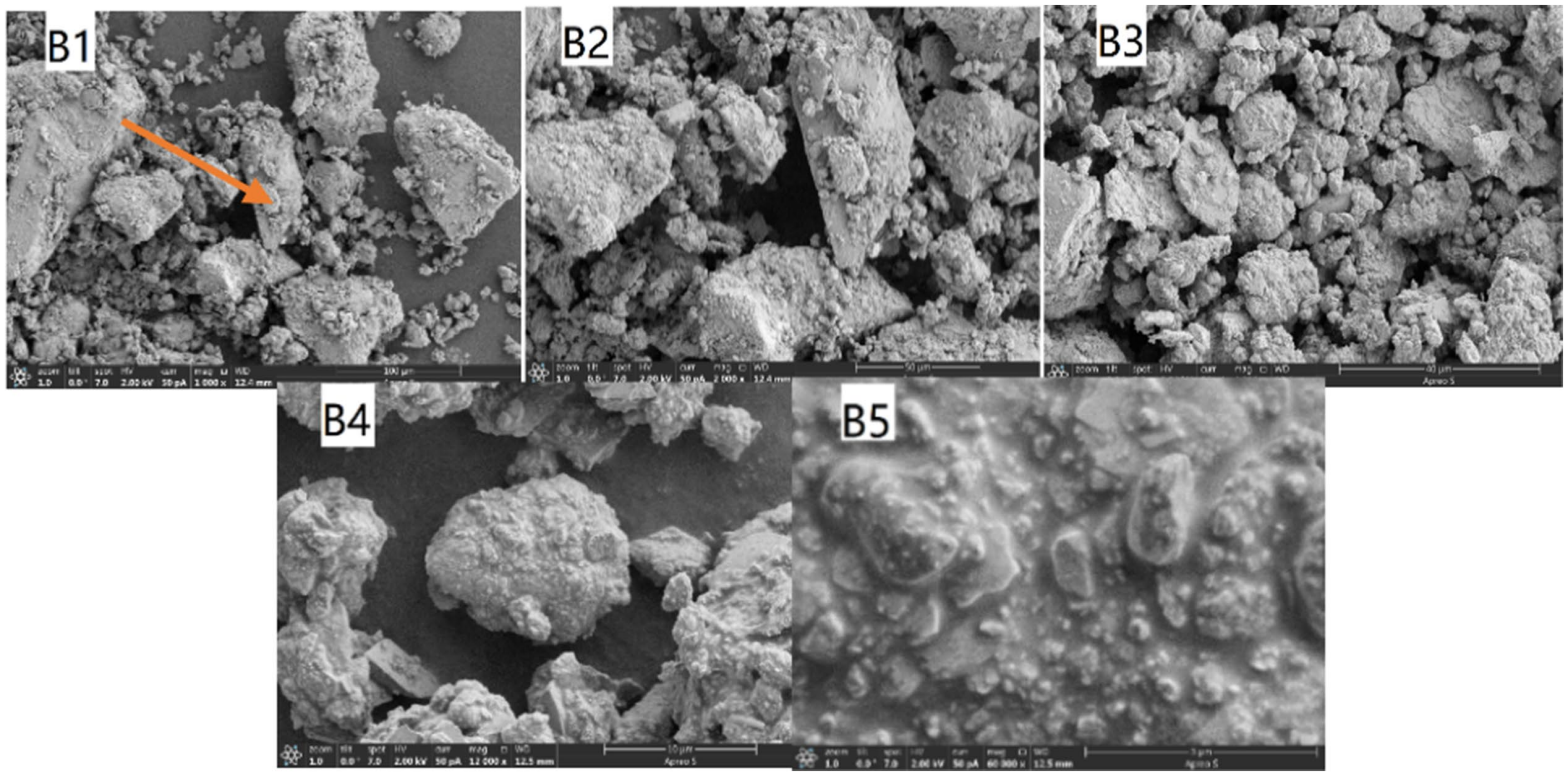
SEM image of cement mixed with capsules.

**Figure 19:**
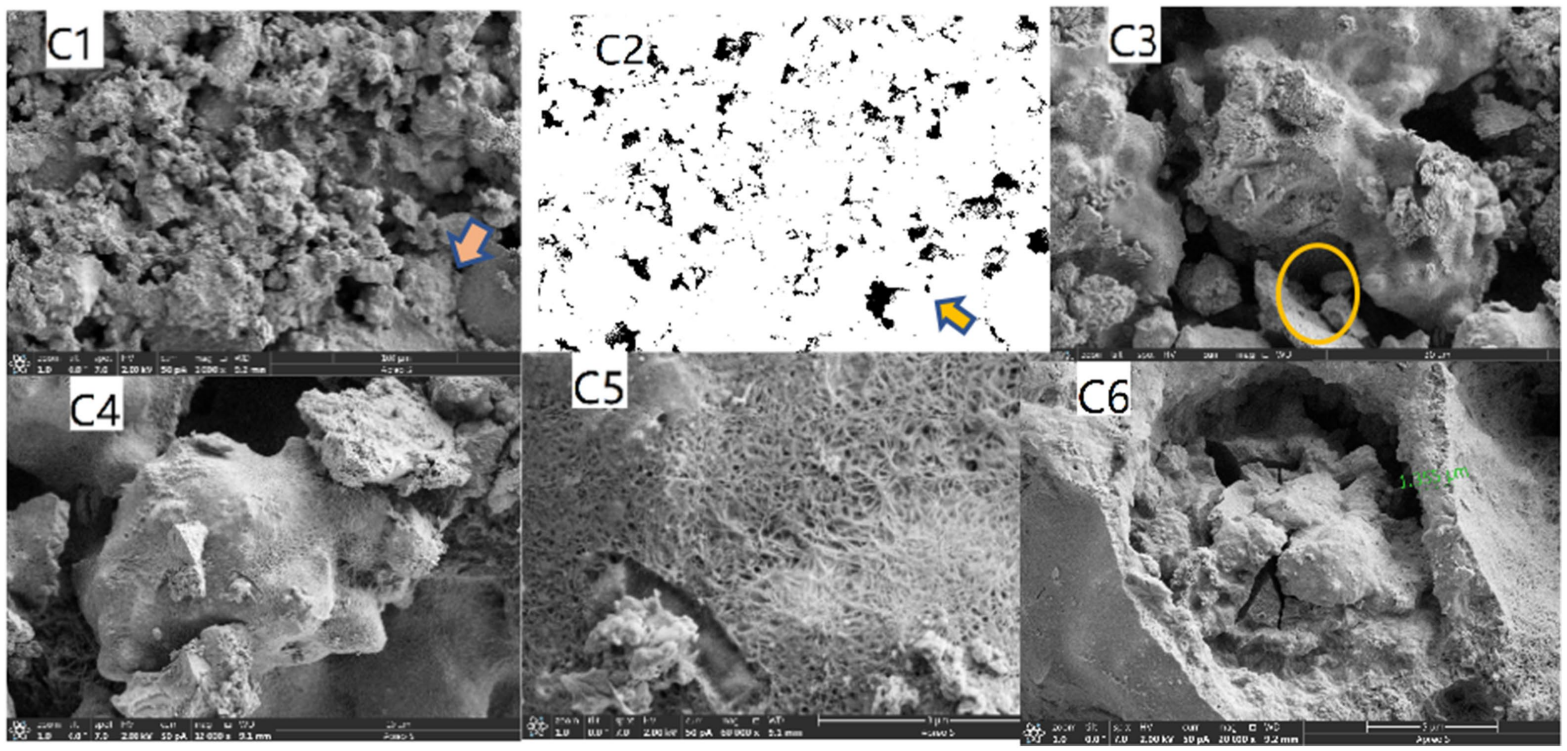
SEM images of the sample sections.

Cement mortar samples with different strength grades exhibit variations in pore size distribution and porosity. Higher-strength samples have smaller pore sizes and lower porosities. SEM was employed to examine the microstructures of precipitates produced by *Bacillus pasteurii*, microcapsule-incorporated grouts, and self-healing microbial concrete sections. The results are presented in Figures 17–19, respectively.

Figure 17 shows *Bacillus pasteurii* and its precipitates. The arrows indicate the bacteria and the metabolically produced calcium carbonate crystals, which are identified as vaterite and aragonite on the basis of their morphology (Zhang et al., 2022). The crystals exhibit mutual cementation, with the microorganisms encapsulated by their secretions.

A2 presents the calcium carbonate secretions magnified 60,000 times.

A3 displays the morphology after bacterial division and detachment of the calcium carbonate shell, where spherical and scattered rod-shaped structures correspond to vaterite and aragonite.

A4 shows the calcium carbonate secretions in vaterite form.

In Figure 18, B1 shows calcium carbonate crystals secreted by *Bacillus pasteurii* attached to hardened clusters within the cement mortar. B2 is a magnified view of the arrow-indicated area in B1, revealing secretions adhering to flaky sand grains. B3 demonstrates that the secretions primarily consist of vaterite. B4 displays a composite image of cement paste mixed with bacterially precipitated calcium carbonate. B5 presents a hydrated mixture of cement paste and bacterial calcium carbonate, where crystals remain attached to the surface of particulate matter.

Figure 19 presents the following microstructural observations:

C1 shows a 1,000 × magnified cross-sectional view of the cement, revealing its inherent microstructure.

C2 is the binarized (ImageJ-processed) version of C1, where pores appear as distinct black regions.

C3 demonstrates that cementitious clusters are interconnected by tobermorite crystals (formed through hydration) and microbially secreted calcium carbonate.

C5 provides a magnified view of the arrow-marked area in C4, clearly displaying crystalline fibrous networks formed by cement hydration.

C6 highlights a crack (arrow-indicated) that appears bridged by calcium carbonate crystals (likely secreted by *Sporosarcina pasteurii* vegetative cells, as referenced in A1) and hydration-derived fibers. The orange-circled zone shows tight, pore-free filling by microbial secretions at the interface.

These findings align with the previously discussed MICP surface treatment effects: reduced capillary water absorption due to pore occlusion. Decreased breathability from the densified microstructure.

On the basis of the analysis of pore-related parameters in self-healing mortar and the microscopic morphology of microbial self-healing concrete, the following conclusions can be drawn:

1. When microbial capsules are incorporated into ordinary cement mortar, the paste becomes more viscous and stable, which is beneficial for concrete formation.
2. During the formation of MICP blocks in these pores, microbial vegetative cells act as nucleation sites for calcium carbonate crystallization. Over time, the number of crystal particles gradually increases, and the particles eventually accumulate, forming robust calcium carbonate blocks that fill cracks and tightly bond the secretions with the concrete matrix.
3. Through microbial metabolism, different types of calcium carbonate crystals are produced under various conditions. These crystals disperse within the microbial mortar, mutually fuse, and hydrate, thereby altering the pore structure. This leads to reduced porosity and significantly enhances the strength of microbial self-healing concrete.

## 4 Conclusion

This study investigated the factors influencing the germination of *Bacillus pasteurii* spores for microbial self-healing concrete applications. The determination of optimized germination conditions for *Bacillus pasteurii* spores, coupled with an emphasis on the synergistic interactions among key parameters, represents a notable innovation. Comparative analysis of the rheological properties between microbial mortar and conventional mortar reveals that the incorporation of microbial capsules endows the microbial mortar with elevated yield stress and composite viscosity. Furthermore, the permeability fractal dimension and backbone fractal dimension of self-healing concrete samples are comparable to those of traditional materials, with the specific range of pore sizes predominantly susceptible to environmental erosion clearly identified. The precipitation and integration of calcium carbonate crystals within the cementitious matrix, along with the consequent increase in the mechanical strength of microbially self-healing concrete, offer novel insights into the underlying healing mechanisms at the microscopic scale.

The key findings and implications are summarized as follows:

1. Optimized germination parameters: The germination of *Bacillus pasteurii* spores was maximized under the following optimized conditions: a microcapsule concentration of 2 g/L, pH 8, and an inosine concentration of 1 g/L. An alkaline environment is conducive to the physiological activity of bacteria. Crucially, Ca^2+^ ions had no inhibitory effect on spore germination, and a thermal stimulus lasting 3 min effectively triggered the germination process.
2. Pore structure characteristics: Mercury intrusion porosimetry analysis revealed that the self-healing concrete samples exhibited a permeability fractal dimension of 2.832 and a backbone fractal dimension of 2.306. These values align closely with those of conventional cementitious materials, demonstrating that the incorporation of microbial healing agents did not significantly alter the inherent complexity of the concrete pore structure. Environmental pore erosion was predominantly concentrated within the pore size range of 300–10,000 nm.
3. Microstructure and Healing Mechanism: SEM analysis confirmed the precipitation of calcium carbonate crystals, primarily identified as vaterite and aragonite, by *Bacillus pasteurii*. Importantly, these bacterially induced crystals effectively integrated and bound within the cementitious matrix, cocrystallizing and fusing with hydration products, including tobermorite. This synergistic interaction resulted in a denser microstructure, reduced overall porosity, and consequently, increased the mechanical strength of the microbial self-healing concrete.
4. Enhanced matrix stability: The incorporation of microcapsules containing *Bacillus pasteurii* within the mortar matrix contributed to improved stability during the cement setting and hardening processes.
5. Future research directions: While this study established optimal germination parameters under controlled conditions, further exploration is warranted regarding the influence of complex physicochemical factors, mimicking the actual internal concrete environment, on spore germination kinetics. Additionally, developing bacterial gradient acclimatization strategies represents a crucial avenue for enhancing the long-term viability and environmental adaptability of *Bacillus pasteurii* within concrete. Further investigations into the precise mechanisms governing pore structure refinement and crack sealing efficacy under realistic service conditions are also recommended.
6. Limitations or Potential Drawbacks.

### 4.1 Simplification of experimental conditions

The experiments in this study were carried out under relatively ideal laboratory conditions, which are quite different from the complex actual environment. For example, the temperature, humidity, and external force in the laboratory are often kept stable, but in actual engineering, these factors fluctuate greatly. This simplification may lead to the situation that the experimental results are better than the actual application effect, and the applicability of the technology in complex environments needs to be further verified.

### 4.2 Insufficiency in cost–benefit analysis

The current research mainly focuses on the performance of the technology, but there is a lack of in-depth analysis of its cost–benefit. The preparation process of the core materials, the construction cost in engineering applications, and the maintenance cost in the later period are all factors that affect the promotion of the technology. If the cost is too high, even if the performance is excellent, it is difficult to be widely used in practical engineering.

### 4.3 Lack of long-term bacterial activity and safety studies

The study focused mainly on determining the optimal germination conditions and short-term effects on pore structure and mechanical properties. However, the long-term activity of *Bacillus pasteurii* in concrete over the service life of the structure was not comprehensively evaluated. There is a need to understand how the bacteria will survive and maintain their self-healing ability over years or even decades. Moreover, potential safety concerns regarding the use of bacteria in concrete, such as the possible release of harmful by-products or the impact on the surrounding environment if the bacteria were to migrate out of the concrete, were not addressed. These aspects are important for the widespread acceptance and application of microbial self-healing concrete technology.

## Data Availability

The original contributions presented in the study are included in the article/supplementary material, further inquiries can be directed to the corresponding author.
